# How can we improve the use of essential evidence-based interventions?

**DOI:** 10.1186/1742-4755-11-69

**Published:** 2014-09-12

**Authors:** José M Belizán, Natasha Salaria, Pilar Valanzasca, Michael Mbizvo

**Affiliations:** Institute for Clinical Effectiveness and Health Policy (IECS), Buenos Aires, Argentina; BioMed Central, London, UK; University of Zimbabwe, Harare, Zimbabwe

## Abstract

Between 250,000-280,000 women die worldwide during pregnancy and childbirth each year and children in low- and middle-income countries are 56 times more likely to die before the age of 5 than children in high-income countries. This Editorial discusses the publishing of a supplement within *Reproductive Health* titled Essential interventions for maternal, newborn and child health which aims to provide a scientific basis to the recommended interventions along with implementation strategies and proposed packages of care.

Clinicians providing care have seen in a relatively short period of time, a positive change in the scientific literature available to support their clinical practice. Inspired by many leaders, the concept of evidence-based interventions fortunately permeates among health care providers, policymakers and even the public. The inspiration and dedicated work of Iain Chalmers, who was instrumental in the development and consolidation of The Cochrane Library, has led to the best compilation of evidence-based care of our time. With the existence of this knowledge hub, we know what works and what does not work in healthcare and medicine. We are also aware of the uncertainties and gaps on the effect of many health care interventions.

Many initiatives have shown that the implementation of several evidence-based interventions could result in a significant reduction of maternal, neonatal and child mortality [1–3]. However, the last report of the Countdown initiative of 2014, in 74 countries, shows unfinished business in achievement of high, sustained, and equitable coverage of essential interventions (EI), showing that the greatest coverage gaps are in family planning, interventions addressing newborn mortality, and case management of childhood diseases [4]. A result of interest from this analysis is that countries with higher levels of intervention coverage tend to have lower levels of child mortality [4]. The low use of evidence-based practices is not only explained by lack of resources, since low use of evidence-based practices is also seen in hospitals of high complexity where resource needs are fulfilled [5–7]. Then, the poor implementation of these beneficial evidence-based interventions can be a consequence of both social inequity and provider behavior. So, can we say that the provision of such interventions can be seen as a human right? In fact, there are some examples of national laws where the provision of evidence-based practices during labor is seen as a woman’s right [8].

Contribution of research is still needed to develop and systematically test interventions aiming to facilitate the implementation of the EI. An emerging initiative, implementation science, needs the strong commitment of many players to guide the application and uptake of proven interventions.

We are very pleased that *Reproductive Health* is publishing a supplement providing scientific basis to the EI. It aims to complement the previous “Born too Soon” supplement [9] in order to accelerate achievement of the Millennium Development Goals 4 and 5, in the low- and middle-income countries (LMIC), where the burden of maternal and newborn mortality and morbidity are the greatest. This initiative, led by WHO, provides a list of evidence-based interventions that have shown significant benefits on reproductive and child health and their application would improve the health outcome of mothers and their children. The articles of this supplement show a diversity of strategies to apply the EI, as delivered through the health sector, from the community up to the referral level of health service provision.

These evidence-based essential interventions are centered on pre-pregnancy, pregnancy, childbirth, postnatal and child health and were classified in categories based on their efficiency [10]. Many interventions can be provided jointly through the implementation of these packages [11] and proposed packages of care [12].

Estimates from this supplement show that between 250,000-280,000 women die worldwide during pregnancy and childbirth each year and that children in LMIC are 56 times more likely to die before the age of 5 than children in high-income countries [13]. Articles in this supplement discuss interventions to decrease such death rates, such as family planning. Family planning is one of the existing coverage gaps reported in the Countdown initiative [4], and it has been observed that the majority of unsafe abortions that occurred in 2008 worldwide (21.6 million), took place in LMIC. Other reported essential interventions during pregnancy could not only prevent maternal deaths, but also improve neonatal morbidity by planning pregnancies at appropriate intervals, helping to manage unplanned pregnancies, prevent and manage STIs such as HIV and PMTCT, and introduce home visits for women and children across the continuum of care [14].

Essential childbirth and postnatal interventions can be preventive and therapeutic. The majority of maternal and neonatal mortality is due to complications during birth. It is known that in LMIC most of the births occur in homes where access to adequate care at delivery is difficult. The presence of a skilled birth attendant is a key low-tech and low-cost opportunity for those in LMIC, along with continuous social support, postnatal and maternal care delivery and community based education and health promotion workshops, which are all associated with positive outcomes. It is also estimated that practicing early initiation of breastfeeding in developing countries, can save up to 1.45 million lives annually [15]. The role of community based delivery strategies to increase access to needed care is vital to generate a change in developing countries.

This supplement also reviews essential interventions for improved child health. From all worldwide deaths estimated for 2011, 80% of the world’s under-five deaths belong to just 25 countries (and only five countries account for half of those deaths: India, Nigeria, Democratic Republic of the Congo, Pakistan and China). Most deaths and disabilities in children under five particularly depend on the management of infectious diseases and this supplement discusses interventions such as exclusive breastfeeding, routine immunizations and vaccinations for children and preventative zinc and vitamin A supplementation in children as interventions associated with a decrease in mortality and disease rates in children [12]. This supplement also shows that high coverage of an expanded immunization program is one of the most cost-effective interventions for reducing mortality and morbidity in this segment of the population. Therefore, it is key that health professionals and policy makers are aware that a great part of the solution depends on the adequate allocation of the available resources and the implementation of cost-effective interventions or packages [14]. In summary, there is a lot of information showing the benefits that the deployment of the EI would have in the population. Some countries have shown that such deployment can be reached and also there are some studies showing successful interventions to change provider’s behavior towards the implementation of evidence-based practices [4, 15]. All the actors in the maternal and child arena must be committed to achieve such a task. National and health authorities must be accountable in taking action to achieve the goals of mortality reduction. Health care providers are major players in this endeavor, including being amenable to their own change on their current practice. A movement in the research arena is needed, that is supported by the financial agencies to help the research teams to focus more on implementation science. Research strategies should be developed and tested aimed at providing efficient interventions to implement the EI and to assess their effectiveness on health outcomes. These strategies should also look for the quality of care when these interventions are deployed. Of major importance in this process is the monitoring/review/audit of practices and taking actions to correct problems to improve coverage with quality. Social organizations should advocate more on the rights of the women and children to receive the EI. Social communicators should empower the population with the knowledge of the EI and the rights to receive them.

The joint effort of many players would significantly improve health and quality of life of mothers, their children and their families.
